# Somatopause, weaknesses of the therapeutic approaches and the cautious optimism based on experimental ageing studies with soy isoflavones

**DOI:** 10.17179/excli2017-956

**Published:** 2018-03-21

**Authors:** Vladimir Z. Ajdžanovic, Svetlana Trifunovic, Dragana Miljic, Branka Šošic-Jurjevic, Branko Filipovic, Marko Miler, Nataša Ristic, Milica Manojlovic-Stojanoski, Verica Miloševic

**Affiliations:** 1 Department of Cytology, Institute for Biological Research "Siniša Stankovic", University of Belgrade, Belgrade, Serbia; 2 Clinic for Endocrinology, Diabetes and Diseases of Metabolism, Clinical Center of Serbia, Faculty of Medicine, University of Belgrade, Belgrade, Serbia

**Keywords:** somatopause, therapy, experimental ageing, soy isoflavones

## Abstract

The pathological phenomenon of somatopause, noticeable in hypogonadal ageing subjects, is based on the growth hormone (GH) production and secretion decrease along with the fall in GH binding protein and insulin-like growth factor 1 (IGF-1) levels, causing different musculoskeletal, metabolic and mental issues. From the perspective of safety and efficacy, GH treatment is considered to be highly controversial, while some other therapeutic approaches (application of IGF-1, GH secretagogues, gonadal steroids, cholinesterase-inhibitors or various combinations) exhibit more or less pronounced weaknesses in this respect. Soy isoflavones, phytochemicals that have already demonstrated the health benefits in treated elderly, at least experimentally reveal their potential for the somatopausal symptoms remediation. Namely, genistein enhanced GHRH-stimulated cAMP accumulation and GH release in rat anterior pituitary cells; refreshed and stimulated the somatotropic system (hypothalamic nuclei and pituitary GH cells) function in a rat model of the mild andropause, and stimulated the GH output in ovariectomized ewes as well as the amplitude of GH pulses in the rams. Daidzein, on the other hand, increased body mass, trabecular bone mass and decreased bone turnover in the animal model of severe andropause, while both isoflavones demonstrated blood cholesterol-lowering effect in the same model. These data, which necessarily need to be preclinically and clinically filtered, hint some cautious optimism and call for further innovative designing of balanced soy isoflavone-based therapeutics.

## Introduction

Ageing represents a complex phenomenon characterized by the temporal, social and psychophysiological dimension. While the temporal one is general and intuitively clear, reflecting the time flow, the two other dimensions are interconnected and have some gender-related specificities. Female ageing is outlined by initial continuity of the process, leading to the fertility-related milestone - the menopause, a “point of discontinuity“ or the specific life transition associated with an increase in various health risk factors including cardiovascular issues, osteoporosis, diabetes or cancer (Perry et al., 2015[114]; Koebele and Bimonte-Nelson, 2016[80]). Some psychological disorders considering low moods, depression, anxiety states, insomnia, changes in cognition or reduction of sexual interest also accompany the post-menopausal period of life (Pearce and Hawton, 1996[113]; Santoro et al., 2015[119]). An abrupt and permanent cessation of the ovarian function is responsible for the menopausal transition (Calabrese et al., 2014[33]; Ajdžanović et al., 2017[4]). Ovarian cell loss begins even prenatally in numerous female mammals, deriving exponential depletion of primary follicles and oocytes, associated with loss of fecundity by midlife (Finch, 2014[53]). In the post-menopause, follicle-stimulating hormone levels are significantly higher, estradiol levels are fallen, while inhibin B and anti-Mullerian hormone are undetectable (Burger et al., 2007[32]). Reconsideration of the role and purpose in life, personality, interpersonal relationships or body image influence the experience of menopause in the social terms (Deeks, 2004[46]). On the other hand, male ageing is progressive and gradual in its essence, while low free testosterone is the key characteristic of the hormonal status of ageing males (Gray et al., 1991[68]; Chahal and Drake, 2007[37]; Ajdžanović et al., 2015[5], 2016[13]). So far accumulated data indicate ageing-induced decrease in the Leydig cell steroidogenic capacity with an increase in cyclooxygenase-2 activity and its' tonic inhibition of steroidogenic acute regulatory gene expression; lowered Sertoli cell function and number, decline in seminiferous tubule volume and sperm content, as well as raised germ cells apoptosis rate (Wang et al., 2002[145]; Wang and Stocco, 2005[146]). Diminished endogenous testosterone production, enabling circulating free testosterone lower than 220 pmol/L, grounds a specific multi-symptomatic syndrome known as andropause or late-onset hypogonadism (LOH) (Vance, 2003[140]; Morales, 2004[102]; Singh, 2013[125]). To be precise, primary hypogonadism (testicular failure) as well as the secondary hypogonadism (hypothalamic-pituitary-gonadal axis failure) entirely represent the phenomenon of andropause (Golan et al., 2015[66]). Similarly to the menopausal women, various psycho-social issues like depression, lack of motivation, lower psychological vitality, anxiety, irritability, insomnia, difficulty in concentrating, memory impairment, decreased work performances, low dominance and decreased libido are observable in the ageing men (Amore, 2005[18]).

The neuroendocrine regulation of the ageing process is attracting intensive attention of the investigators during the current epoch of a dramatic increase in the elderly people share (Jones and Boelaert, 2015[77]). Ageing-induced extinction/dysfunction of the regulatory hippocampal, hypothalamic and limbic neurons and synapses and the compensatory gliosis (Mani et al., 1986[91]; Ferrari et al., 2001[48]) are followed with a decrease of hormonal secretion within majority of the neuroendocrine axes, reduced sensitivity of tissues to hormonal actions and the loss of normal circadian rhythms (Jones and Boelaert, 2015[77]). Additionally to previously mentioned, ageing-related structural and functional disorders of the reproductive system (menopause and andropause) as well as the hypothalamic-pituitary-adrenal (HPA) axis perturbations during ageing (adrenopause) linked with metabolic syndrome and cardiovascular issues, hypogonadal ageing subjects frequently suffer from the loss of growth axis function (somatopause), associated with reductions in skeletal muscle and bone mass/strength (Ferrari and Mantero, 2005[49]; Lombardi et al., 2005[87]; Golan et al., 2015[66]; Jones and Boelaert, 2015[77]). The impression is that the somatopausal perspective of musculoskeletal issues in elderly received insufficient attention within the research circles.

Hormone replacement therapy (HRT) of the ageing subjects appears to be an acceptable medical approach, considering that the irreversible age-related changes at the tissue/organic level almost entirely exclude the causal therapy modalities (Beg et al., 2008[25]; Shifren and Shiff, 2010[123]; Ajdžanović et al., 2015[5], 2016[13]). Significant benefits pertinent to the prevention of menopausal osteoporosis as well as the management of a certain psychological disorders, frequent in this period of female life, are observed upon menopausal estrogen therapy (Al-Safi and Santoro, 2014[17]; Cauley, 2015[35]). In line with this, the affirmative aspects of testosterone application to ageing males have included the prevention or reversion of bone deterioration, glucocorticoid hypersecretion, sexual dysfunction and depression (Myers and Meacham, 2003[105]; Amore et al., 2009[19]; Ajdžanović et al., 2017[3]). Treatment with growth hormone (GH) during ageing was considered as a possible “fountain of youth”, hence as some good solution in improving the functional status of elderly (Clayton et al., 2007[40]; Sattler, 2013[120]). However, keeping in mind the possible harmful effects of estrogen replacement therapy (thromboembolism, breast or endometrial cancer development) or following the testosterone supplementation (prostate cancer development, cardiovascular issues, hepatotoxicity), careful selection of dosage and duration of their use is strongly recommended (Grady et al., 2000[67]; Fournier et al., 2003[55]; Myers and Meacham, 2003[105]; Jockenhövel, 2003[76]; Albert and Morley, 2016[15]). Also, although GH therapy has been shown to exert positive effects in GH-deficient patients, it is known that GH/insulin-like growth factor 1 (IGF-1) deficiency may result in prolonged life expectancy, at least in animals (Giordano et al., 2008[62]). Different authors generally consider the GH treatment as highly controversial, from the perspective of safety, efficacy and its exact role in elderly individuals (Shim and Cohen, 1999[124]; Liu et al., 2007[86]; Giordano et al., 2008[62]).

When it comes to the focus of biomedically-oriented part of the scientific community, the past two decades have been in the sign of massive investigation at the field of plant-derived, preferably safe and effective, alternatives to HRT. A complex array of polyphenolic, bioactive non-nutrients isolated from vegetables, legumes, cereals, fruits or tea, also known as phytochemicals, have demonstrated significant health benefits upon consumption either as an integral part of the food or as a food supplement/alternative remedy (Adlercreutz and Mazur, 1997[1]; Setchell, 1998[122]; Tham et al., 1998[132]; Chanet et al., 2012[38]).

**Figure 1:**
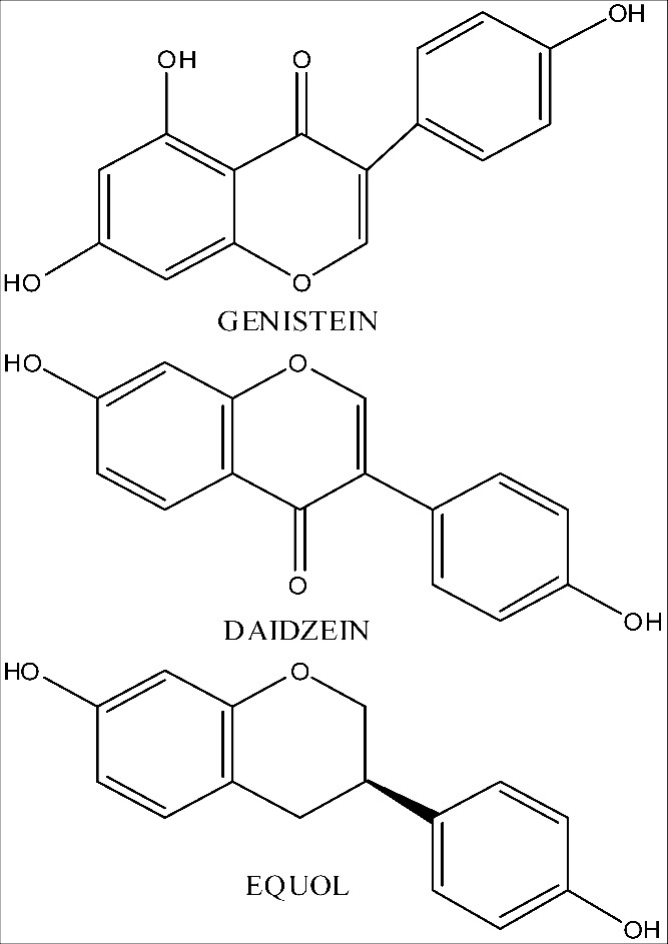
The chemical structures of soy isoflavones

Soy isoflavones (commonly genistein, daidzein and equol - Figure 1(Fig. 1)) represent diphenolic, estrogen-like compounds that may exist without or with glucose residues attached (aglycones or 7-O-β-D glucosides). They perform weak estrogenic and antiestrogenic activity both *in vitro* and *in vivo* (Price and Fenwick, 1985[116]). Also, soy isoflavones manifest tyrosine kinase inhibiting effects (Akiyama et al., 1987[14]) and strong antioxidative activity (Benassayag et al., 2002[26]). Representative studies have shown that some positive outcome could be noticed in soy isoflavone treatment of breast and prostate cancer, osteoporosis, coronary heart disease, as well as ageing-related psychological symptoms (Casini et al., 2006[34]; Messina, 2010[93]; Messina et al., 2010[94]; Andres et al., 2011[20]). The research experience that qualifies us for an credible opinion related to soy isoflavone effects during ageing is based on a multi-year exploitation of different rat models of andropause and menopause, in the context of these compounds application (Ajdžanović et al., 2009[10][11], 2011[12], 2012[9], 2014[8][6]; Milošević et al., 2009[99]; Filipović et al., 2007[52], 2010[50]; Šošić-Jurjević et al., 2007[126], 2010[127], 2012[128], 2014[129]; Pantelić et al., 2013[112]; Trifunović, 2012[137]; Trifunović et al., 2012[134], 2014[135][133], 2016[136]; Medigović et al., 2015[92]). The general scarcity of data concerning the soy isoflavone effects in somatopause, together with the fact that our ageing rat models, besides numerous neuroendocrine axes disturbancies, manifest the somatopausal symptoms also, highlight the reported soy isoflavone role in their, at least partly, alleviation (Trifunović, 2012[137]; Ajdžanović et al., 2014[8]; Trifunović et al., 2014[133], 2016[136]). This analytical text is *inter alia* devoted to detailed, experimentally grounded elaboration of soy isoflavone-mediated effects through the somatotropic *i.e.* GH/IGF-1 system in ageing models.

## Phenomenology of the Somatopause

### Somatotropic system

**Figure 2:**
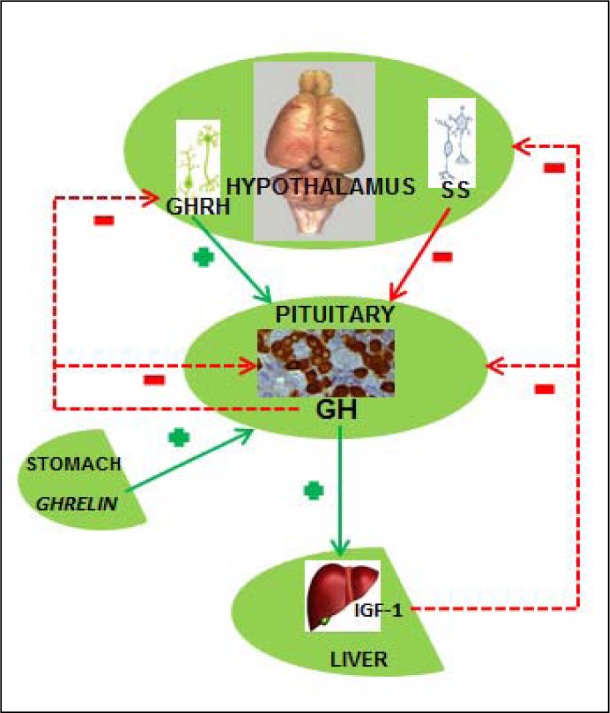
Internal regulation of the somatotropic system functioning

Somatotropic system plays the essential role in the hormonal regulation of postnatal growth and development in mammals. The system covers regulation of the GH (also known as somatotropin) secretion from pituitary somatotropes (GH cells), GH distribution and its actions in peripheral tissues, interaction with the specific GH receptors (GHr), as well as the endocrine, paracrine and autocrine responses, mostly mediated by IGF-1 (Figure 2(Fig. 2)). Hypothalamic regulatory centers, anterior pituitary, peripheral target tissues and different receptors and signal molecules belong to the somatotropic system (Giustina and Veldhuis, 1998[64]; Le Roith et al., 2001[84]). The numerous inter-constituent interactions within the system are modulated by different neurotransmitters and neuropeptides, sex steroids, corticosteroids, thyroxine and some metabolic signals. GH affects a variety of target tissues and organs, like skeletal muscles, bones, liver, gastrointestinal tract, brain, heart, kidneys, mammary glands, ovaries, testes, spleen, thymus, bone marrow and skin (Veldhuis et al., 2005[143]). Somatotropic system plays the crucial regulatory role in protein, carbohydrate and lipid metabolism (Daughaday, 1992[45]), while GH stimulates the liver IGF-1 biosynthesis, thus ensuring the presence of its most important mediator in the physiological action (Haymond and Mauras, 1996[69]; Le Roith et al., 2001[84]). However, in some type of cells, like chondrocytes and mature adipocytes, GH action is IGF-1 independent (Hwa et al., 1999[72]). The somatotropic system activity could be influenced by the environmental, metabolic and hormonal factors, including hunger, obesity, injuries, sexual activity as well as glucocorticoid and thyroid status. These factors affect both hypothalamic regulation of GH production/secretion and the following response of the target tissues (Giustina and Veldhuis, 1998[64]). It should be emphasized that GH is in mammals secreted as a series of pulses. In normal young individuals, a major secretory episode occurs shortly after sleep onset and coincides with the first period of slow-wave sleep (Van Cauter et al., 1998[138]).

The crucial GH secretion regulators are two hypothalamic hormones - growth hormone-releasing hormone (GHRH) and somatostatin (SS). Presence of different GHRH and SS concentrations in the pituitary portal bloodstream leads towards different GH concentrations in the systemic circulation, enabling GH release when portal SS level is low and the related GHRH level is high (Tannenbaum and Ling, 1984[131]; Plotsky and Vale, 1985[115]). The mechanism of GH release, regulated by these two neuropeptides, involves adenylate cyclase/cAMP pathway and the intracellular Ca^2+^ changes (Bilezikjian and Vale, 1983[28]; Frohman and Jansson, 1986[57]; Lussier et al., 1991[89]). Increase of the cAMP levels triggers the Na^+^ channels opening, membrane depolarization and Ca^2+^ influx *via* the L-type of Ca^2+^ channels (Lussier et al., 1991[90]; Naumov et al., 1994[106]). The increase of intracellular Ca^2+^ is the basis of GH release, realised over the exocytosis process. Production of cAMP and GH release also depends on some additional signal mechanisms, including protein kinase C, tyrosine kinase, diacyglycerol and phospholipase (Cronin et al., 1986[44]; Ogiwara et al., 1997[109]). In line with this, there are some data decribing GHRH influence on GH gene transcription through cAMP-dependent process (Clayton et al., 1986[41]).

The third hormone involved in the regulation of GH secretion is 28 amino acids-containing acylated peptide ghrelin (van der Lely et al., 2004[139]). It is dominantly produced in stomach, while some considerably lower concentrations of the hormone are detected in guts, pancreas, kidneys, lungs, immune system, placenta, testes, pituitary and hypothalamus (Korbonits et al., 2001[82]; Lu et al., 2001[88]). Ghrelin stimulates GH release synergistically with GHRH, after binding to GH-secretagogue receptors (Kojima et al., 1999[81]). Somatomedin- (especially IGF-1) as well as the own GH-negative feedback additionally control GH release (Giustina and Veldhuis, 1998[64]).

## General Considerations of Somatopausal Symptoms and the Morphophysiological Basis of Somatopause

In the advanced period of life, production and secretion of GH decrease in parallel with the fall in GH binding protein and IGF-1 levels, all of which is often encircled using the complex term - somatopause (Sattler, 2013[120]; Junnila et al., 2013[78]). Actually, production of GH was far ago reported to decline by 14 % *per* life decade (Iranmanesh et al., 1991[74]). While pubertal boys produce 1.0-1.5 mg/day of GH, in aged men this hormone level is around 50 μg/day (Veldhuis et al., 1995[142]). During middle-age and after that period 35 % of men were found to be GH deficient (Rudman et al., 1990[118]). Basically, the contribution of sleep-dependent GH release to the daily output in women is lower and more variable than in men (Van Cauter et al., 1998[138]). It was observed that the mean GH release and pulse amplitude are significantly lower in aged, unrestrained female rhesus monkeys than in young female individuals (Woller et al., 2002[147]). In women, the fall in GH release is amplitude- rather than frequency-modulated and it was linked to estrogen status (Ho and Weissberger, 1990[71]). Somatopause is regularly followed with reductions in skeletal muscle and bone mass/strength (sarcopenia, osteopenia or osteoporosis), weakness, frailty, loss of physical functions and vitality, central adiposity, cardiovascular risks and deterioration of mental function (Lombardi et al., 2005[87]; Sattler, 2013[120]).

Mechanisms responsible for the age-dependent instability of GH release include both peripheral influences (gonadal steroid levels, adiposity) and, more important, changes in hypothalamic neuropeptides and neurotransmitters entwined with decreased GHRH secretion as well as SS hypersecretion (Müller et al., 1995[103]; Ghigo et al., 1996[59]; Arvat et al., 1999[22]; Veldhuis et al., 2002[141]). Giordano et al. (2005[63]) point out that the hypofunction of somatotropic system in ageing represents the impressive example of decreased activity as a consequence of age-related changes in the neural control of GH cells. It shouldn't be overlooked that the age-related variations in a natural GH secretagogue ghrelin effects could play an important role in the decreased GH secretion in elderly subjects (van der Lely et al., 2004[139]; Arvat et al., 2000[21]).

### Somatopause through the lens of animal studies

In order to precisely and consistently position the phenomenon of somatopause in the context of animal studies, we need some broader perspective of the GH/IGF-1 deficiency models analyze. Namely, while the creation of various knockout animal models enables general investigation of the GH/IGF-1 deficiency consequences, somatopause *per se* is usually being identified in parallel with the other morphophysiological signs of deterioration in the animal models of ageing.

Low GH and IGF-1 levels characterize specifically genetically modified invertebrates *Caenorhabditis elegans* and *Drosophila melanogaster* used for the ageing studies, as well as laboratory rodents known as lit/lit mice, Ames dwarf, Snell dwarf, Lewis dwarf or Laron dwarf (Junnila et al., 2013[78]; Sattler, 2013[120]). Knockout of prop-1 producing Ames dwarfs and Pit-1 producing Snell dwarfs results in GH/IGF-1 deficiency (Brown-Borg et al., 1996[31]; Flurkey et al., 2001[54]). Pit-1 encodes the protein necessary for the differentiation of pituitary GH, thyroidstimulating hormone (TSH) and prolactin cells, so Snell mice lack all three hormones (Li et al., 1990[85]). Similarly, GH-deficient Ames dwarf mice possess mutation in the prop-1 gene responsible for the expression of 'homeobox protein prophet of Pit-1' (Junilla et al., 2013[78]). On the other hand, Laron dwarf mice manifest GH receptor mutation and are GH-resistant, with decreased IGF-1 levels (Coschigano et al., 2003[43]). It should be highlighted that dwarf animal models, with decreased tone of the somatotropic system, have extended longevity (Junnila et al., 2013[78]). They were proposed to be protected from cancer and *diabetes mellitus*, two widely spread ageing-related morbidities, due to the affection of critical cellular regulatory systems involved in response to stress, metabolism or energy balance (Junnila et al., 2013[78]; Sattler, 2013[120]). Interestingly, the Kopchick laboratory experience with the lines of mice suffering from targeted GHr deletion, limited to the specific tissues (adipose, liver, skeletal or cardiac muscle), indicates the lack of global GHr deletion effects on longevity (Bartke et al., 2016[24]).

Herein, we have no intention to polemicize with the argumented attitude pertinent to the longevity of GH/IGF-1 deficient animal models, but tend to emphasize the somatopause-related indicators in the animal ageing models, attributable to the quality of life. Our accumulated experience in the field of experimental ageing results from the exploitation of Wistar rat models of andropause and menopause. Considering the importance of the 'stereology approach', as essential in the discovery of important concepts in the variety of cell biology investigations (von Bartheld and Wouters, 2015[144]), the stereological counting in our laboratory is performed using microscopy on the histological tissue sections, as a method of semi-automated, computer-based microscopy systems (newCast, Visiopharm, Denmark). Before elaboration of detected degenerative changes of the somatotropic system, present in these models, we will respectively describe its normal features in adult rats used as the controls, as well as the features of somatotropic system in the ageing controls.

**Figure 3:**
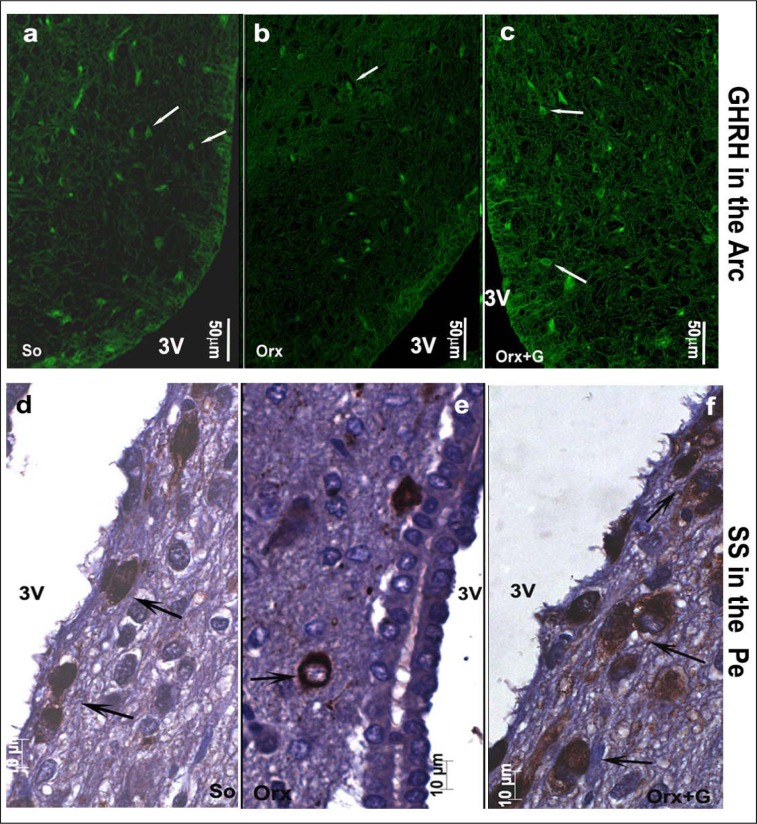
Somatotropic system hypothalamic regulatory neurons in: a, d) So - sham operated, b, e) Orx - orchidectomized and c, f) Orx+G - genistein treated orchidectomized adult male rats (Trifunović, 2012; Trifunović et al., 2016). GHRH - growth hormone-releasing hormone neurons, SS - somatostatin neurons, Arc - hypothalamic arcuate nucleus, Pe - hypothalamic periventricular nucleus. White and black arrows are directed towards the representative GHRH and SS neurons. Orchidectomized adult male rats represent animal model of the mild andropause.

**Figure 4:**
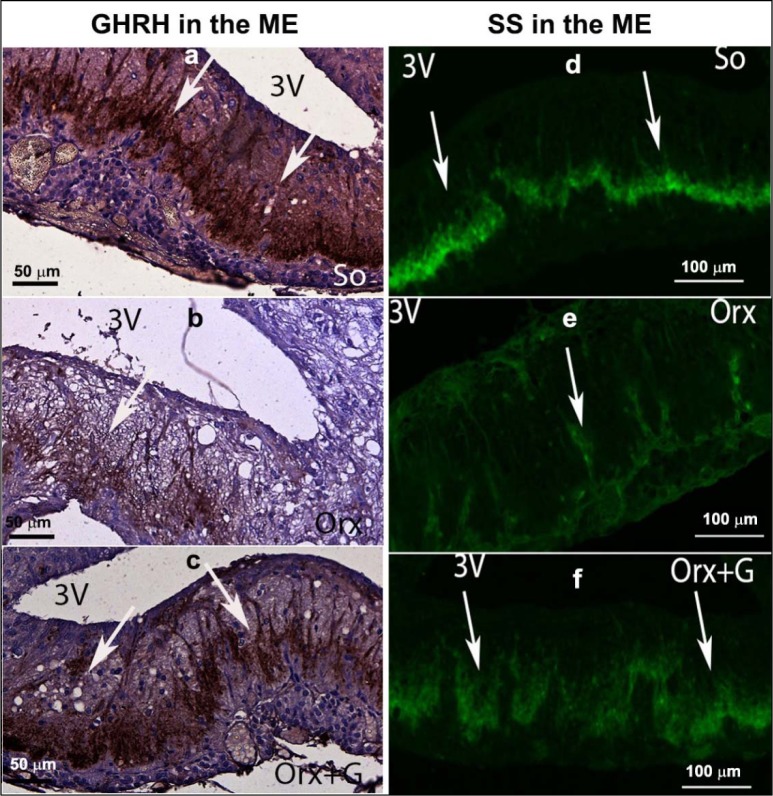
Somatotropic system hypothalamic regulatory neurons in: a, d) So - sham operated, b, e) Orx - orchidectomized and c, f) Orx+G - genistein treated orchidectomized adult male rats (Trifunović, 2012; Trifunović et al., 2016). GHRH - growth hormone-releasing hormone neurons, SS - somatostatin neurons, ME - median eminence. White arrows indicate the representative GHRH and SS immunoreactivity.

**Figure 5:**
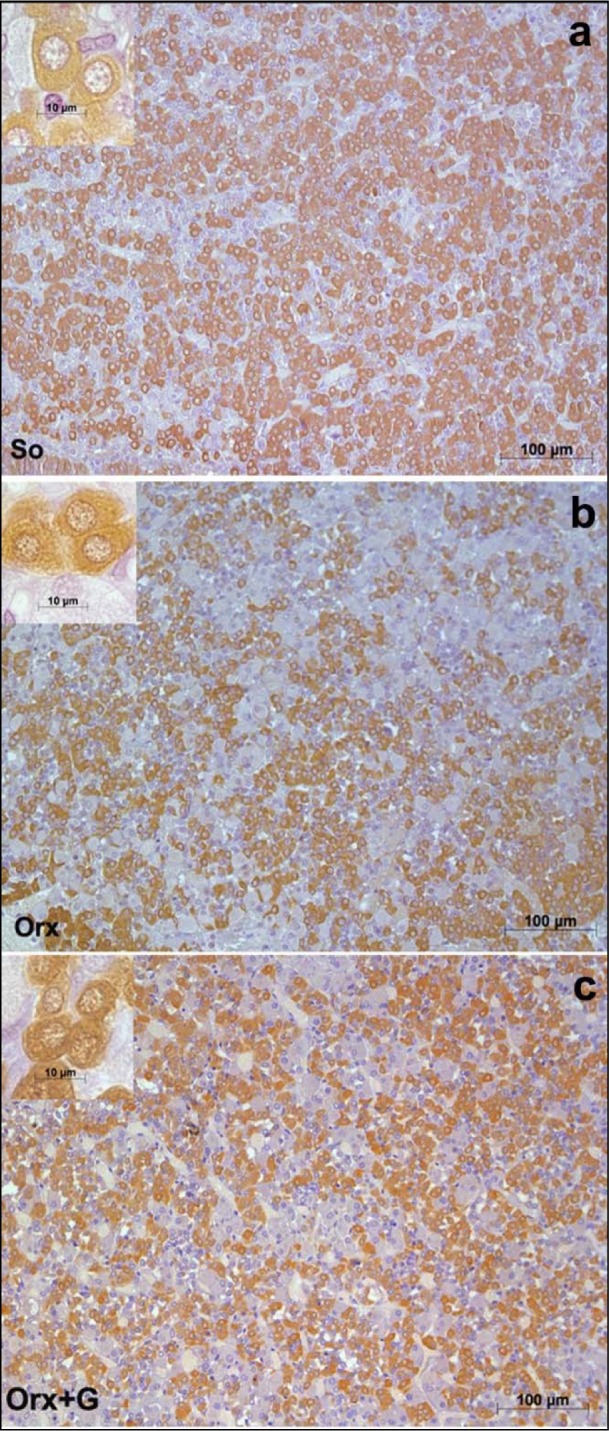
Immunolabeled GH cells in: a) So - sham operated, b) Orx - orchidectomized and c) Orx+G - genistein treated orchidectomized adult male rats (Trifunović, 2012; Trifunović et al., 2014b).

In the first instance, we will focus on adult male rats that have preserved central regulation of endocrine processes and provide the control values for comparison with the adequate ones after orchidectomy (orchidectomized adult male rats represent animal model of the mild andropause). The mean body mass of sham-operated, control adult male rats in our experiment amounted 362.4 ± 36.3 g (Trifunović, 2012[137]). The average absolute pituitary weight was 12.9 ± 1.1 mg, while the average pituitary volume was 5.5 ± 0.6 mm^3^, in the same animals (Trifunović, 2012[137]; Trifunović et al., 2014[133]). GHRH immunoreactive neurons, located in the ventrolateral portion of the hypothalamic arcuate (Arc) nucleus, demonstrated a clear immunofluorescent staining characteristics in sham-operated adult male rats (Figure 3a(Fig. 3)) (Trifunović, 2012[137]; Trifunović et al., 2016[136]). SS immunoreactivity, expressed in the neurons located within a discrete zone of the hypothalamic periventricular (Pe) nucleus, alongside the third brain ventricle, is also found to be distinctive in the same group of rats (Figure 3d(Fig. 3)) (Trifunović, 2012[137]; Trifunović et al., 2016[136]). Besides these two specified hypothalamic nuclei, GHRH and SS noticeable immunoreactivities were likewise identified in the median eminence (ME), a structure at the base of the hypothalamus, of our control adult male rats (Figure 4a,d(Fig. 4)) (References in Figure 4: Trifunović, 2012[137]; Trifunović et al., 2016[136]). The average volume of Arc nucleus amounted 0.175 ± 0.017 mm^3^ while the same parameter of Pe nucleus was 0.259 ± 0.014 mm^3^, in sham-operated adults (Trifunović, 2012[137]; Trifunović et al., 2016[136]). In line with this, volume density of GHRH neurons in Arc nucleus and volume density of SS neurons in Pe nucleus were around 14% and 7.9 ± 0.8%, respectively (Trifunović et al., 2016[136]). GH cells are the predominant cell phenotype in the anterior pituitary of adult male rats (Takahashi, 1992[130]; Milošević et al., 1998[96]; Milošević, 2001[95]). In our male controls, immunohistochemically labelled GH cells varied from round or ovoid to pyramidal and ellipsoid in shape, and possessed a large, spherical, eccentrally located nucleus (Figure 5a inset(Fig. 5)) (Milošević et al., 1998[96]; References in Figure 5: Trifunović, 2012[137]; Trifunović et al., 2014[133]). The cell distribution was even and considered either the presence of clusters or a single cell between capillaries, in the pituitary *pars distalis* (Figure 5a(Fig. 5)) (Milošević et al., 1998[96]; Trifunović, 2012[137]; Trifunović et al., 2014[133]). The mean value of GH cell volume density amounted 23.8 ± 0.87 %, while the numerical density of GH cells was 16.4 ± 1.4 x 10^4^ mm^-3^, in sham-operated male rats (Trifunović, 2012[137]). The total number of GH cells was 7.0 ± 0.3 x 10^5^ and the GH cell volume amounted 1498.0 ± 109.2 μm^3^, in the same adult group (Trifunović, 2012[137]; Trifunović et al., 2014[133]). Finally, the circulating concentration of GH was 3.6 ± 0.4 ng/ml, in the sham-operated controls (Trifunović, 2012[137]; Trifunović et al., 2014[133]). 

In orchidectomized adult male rats (animal model of the mild andropause), the mean body mass amounted 330.4 ± 32.3 g and was significantly (p<0.05) decreased in comparison with the sham-operated adult controls (Trifunović, 2012[137]). After orchidectomy, the average absolute pituitary weight was 14.9 ± 1.1 mg, which represented the increase when compared to the control value, while the average pituitary volume was 6.1 ± 0.6 mm^3^ and remained almost unchanged (Trifunović, 2012[137]; Trifunović et al., 2016[136]). GHRH immunoreactivity in the Arc nucleus as well as SS immunostaining in the Pe nucleus, of orchidectomized adult males, were less noticeable than in sham-operated controls (Figure 3b, e(Fig. 3)) (Trifunović, 2012[137]; Trifunović et al., 2016[136]). In line with this, the ME-related staining intensities of GHRH and SS neurons were lower (p<0.05) after orchidectomy of adult rats (Figure 4b, e(Fig. 4)) (Trifunović, 2012[137]; Trifunović et al., 2016[136]). The volume of the Arc nucleus was 33 % (p<0.05) larger in orchidectomized than that in sham-operated adults, while orchidectomy did not provoke changes of the Pe nucleus volume in relation to the same control group (Trifunović et al., 2016[136]). Upon orchidectomy, the volume densities of GHRH neurons in the Arc nucleus and of SS neurons in the Pe nucleus were decreased (p<0.05) by 22 % and 42 % respectively, in comparison with sham-operated control animals (Trifunović, 2012[137]; Trifunović et al., 2016[136]). The visual impression of GH cell histological appearance in orchidectomized adult rats implied their decreased number and staining intensity, with unchanged shape and size, all related to sham-operated controls (Figure 5b(Fig. 5)) (Trifunović, 2012[137]; Trifunović et al., 2016[136]). After orchidectomy, the mean value of GH cell volume density decreased by 22 % (p<0.05) while their numerical density was almost unchanged, compared to corresponding parameters in sham-operated rats (Trifunović, 2012[137]; Trifunović et al., 2016[136]). The total number of GH cells, their volume as well as the circulating concentration of GH remained insignificantly changed in orchidectomized adult male rats when compared to the adequate values in sham-operated control males (Trifunović, 2012[137]; Trifunović et al., 2016[136]). It can be concluded that the most prominent degenerative changes of the somatotropic system in an animal model of the mild andropause (namely, after elimination of endogenous sex steroids from the hormonal milieu) were observable at the level of hypothalamic regulatory GHRH and SS neurons in Arc and Pe nucleus respectively, as well as in the ME of hypothalamus, while the GH cells suffered of decreased hormone production and the reduction of volume density.

**Figure 6:**
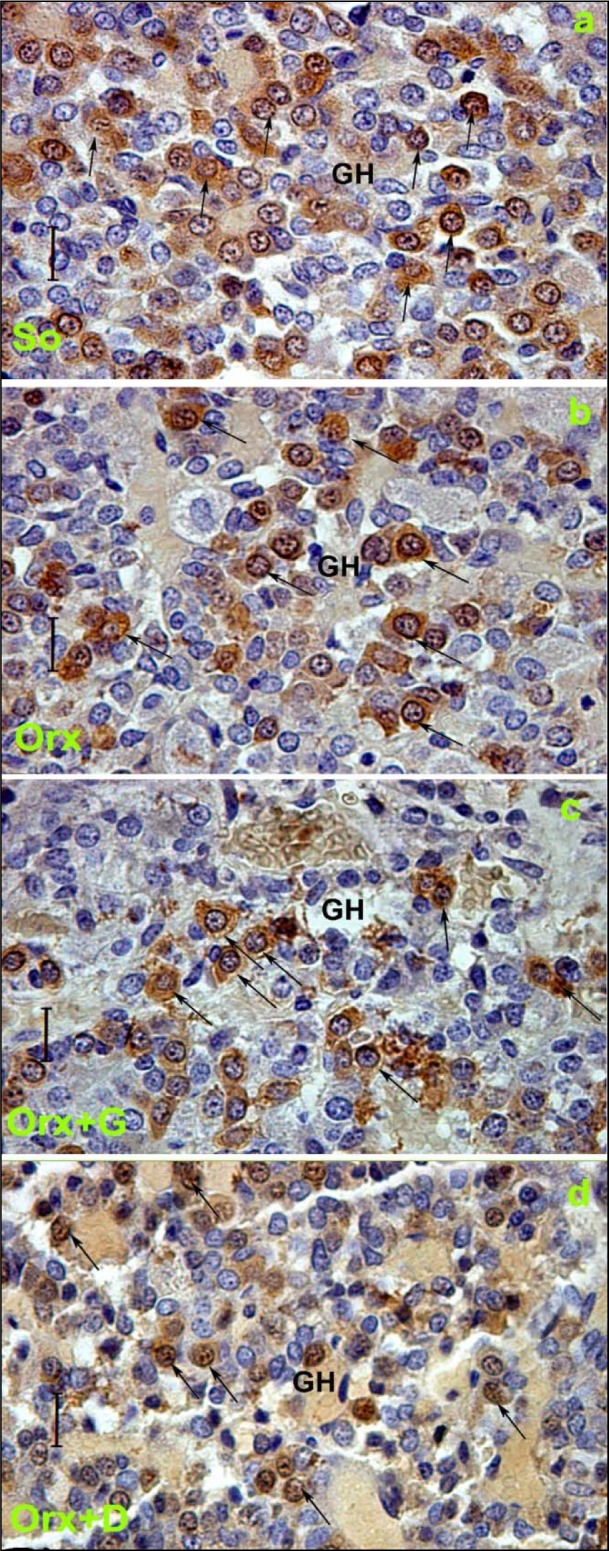
Immunolabeled GH cells in: a) So - sham operated, b) Orx - orchidectomized, c) Orx+G - genistein treated and d) Orx+D - daidzein treated orchidectomized middle-aged male rats; magnification 63x, bar=16 μm (Ajdžanović et al., 2014a). Orchidectomized middle-aged rats represent animal model of the severe andropause.

**Figure 7:**
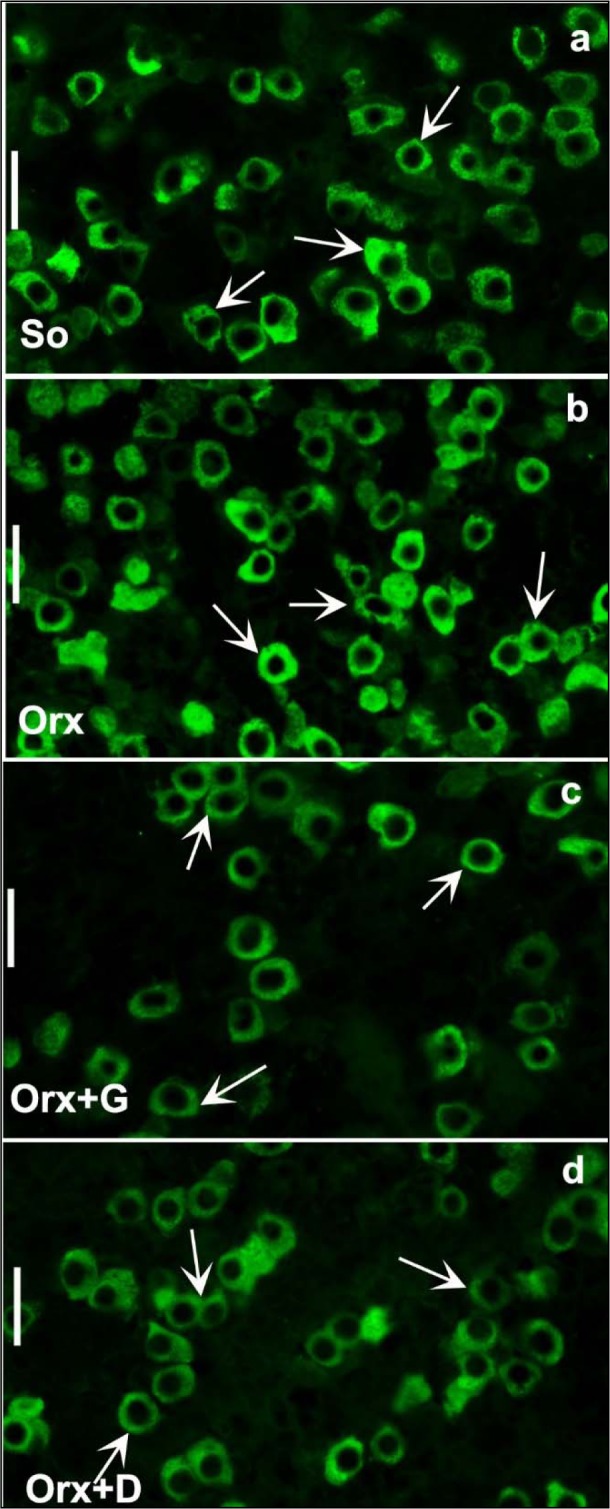
Immunofluorescent appearance of GH cells in: a) So - sham operated, b) Orx - orchidectomized, c) Orx+G - genistein treated and d) Orx+D - daidzein treated orchidectomized middle-aged male rats; bar=20μm (Ajdžanović et al., 2014a).

For the other rat model of the andropause we exploited, orchidectomized middle-aged (16-month-old) male rats - the model of severe andropause, sham-operated middle-aged males provided the values used in the comparison. The mean body mass of middle-aged control males amounted 650.0 ± 29.0 g (Ajdžanović et al., 2014[8]). Their average absolute pituitary weight was 17.0 ± 1.7 mg and the average relative pituitary weight was 2.2 ± 0.1 mg/100 g body mass. Like in sham-operated adult male rat pituitaries, imunopositive GH cells in sham-operated middle-aged males were uniformly distributed (Figure 6a(Fig. 6); Reference in Figure 6: Ajdžanović et al., 2014[8]) in the *pars distalis*, while being completely absent in the pituitary *pars intermedia* (Ajdžanović et al., 2014[8]). We have observed either an individual GH cell or a group of them, especially near the sinusoidal capillaries (Figure 6a(Fig. 6)) (Ajdžanović et al., 2014[8]). Also, the GH cells appeared massive, ovoid to pyramidal in shape, with a spherical, centrally located nucleus (Figure 6a(Fig. 6)) (Ajdžanović et al., 2014[8]). The immunospecific/immunofluorescent signal, reflecting the GH cells hormonal content, was pronounced and widely distributed throughout the cytoplasm (Figure 6a(Fig. 6), 7a(Fig. 7)) (Ajdžanović et al., 2014[8]). The morphometric analysis revealed that a mean GH cell volume amounted almost 600 μm^3^, while their volume density was around 36 %, in sham-operated middle-aged rats (Ajdžanović et al., 2014[8]). It should be noticed that some ageing-induced, somatotropic system-related differences between our adult and middle-aged male controls are visible, especially when it comes to the GH cell volume and volume density.

Our animal model of severe andropause (orchidectomized middle-aged male rats; the central regulation of endocrine processes is disturbed) was characterized by almost 10 % decrease in the mean body mass, when compared to the same parameter in sham-operated middle-aged rats (Ajdžanović et al., 2014[8]). Orchidectomy-caused testosterone elimination from the hormonal millieu of this model did not affect on TSH and thyroxine circulating levels, but significantly increased cholesterol (total, LDL and HDL) blood concentrations (Šošić-Jurjević et al., 2007[126], 2012[128]). While the average absolute pituitary weight was not significantly changed in the orchidectomized model males, the average relative pituitary weight was significantly (p<0.05) increased by 13.6 %, in comparison with the sham-operated controls. There were no observed differences between orchidectomized and sham-operated middle-aged rats when it comes to the GH cell distribution, grouping, shape, nuclei positioning and the immunospecific/immunofluorescent signal (Figure 6b(Fig. 6), 7b(Fig. 7)) (Ajdžanović et al., 2014[8]). Also, the GH cell volume, nuclei volume and volume density were not significantly changed after orchidectomy of middle-aged male rats (Ajdžanović et al., 2014[8]). Obviously, the elimination of circulating sex steroids achieved by orhidectomy did not additionally promote some degenerative changes of the somatotropic system (GH cell morphometric parameters) in middle-aged male rats. In parallel, a series of bone homeostasis-related parameter values measured after orchidectomy indicated the presence of trabecular bone deterioration in this animal model (Filipović et al., 2007[52], 2010[50], 2013[51]; Ajdžanović et al., 2017[3]).

Finally, a few text rows will be given to the somatotropic system-related changes in middle-aged female rats (14-month-old; animal model of the menopause), used in our experimental praxis (Milošević et al., 2000[97], 2005[98]). The mean body mass, as well as the average absolute and relative pituitary weight values were considerably higher in middle-aged females than in adult female controls (Milošević et al., 2000[97], 2005[98]). On the other hand, the morphometric parameters of immunopositive GH cells *i.e.* the cell volume, nuclei volume and volume density, together with circulating GH, have synchronously fallen with ageing of female rats (Milošević et al., 2000[97], 2005[98]). Accordingly, the female ageing rat model is characterized by distinct impairment of the somatotropic system operative component (GH cells) habitus and function, no matter the regular process of a mass/weights rise.

### Clinical recognition of the somatopause and weaknesses of existing therapeutic approaches

Similarly to the situation in an animal model of ageing, where the somatopausal symptoms are identified in parallel with the other morphophysiological signs of deterioration, clinical recognition of the somatopause merges in a wider horizon of degenerative changes occuring with advanced age. Since we have above already mentioned the clinical signs of somatopause, it can be said that ageing-caused (patho)physiological changes within the human body are similar to those observed in GH deficiency, while the GH treatment was considered to be beneficial when it comes to the maximum oxygen consumption and overall life quality (Lombardi et al., 2005[87]; Clayton et al., 2007[40]). However, despite the optimistic thesis, randomized and controlled studies revealed that the effects of GH treatment in healthy elderly subjects actually are less promising (Lamberts, 2000[83]; Lombardi et al., 2005[87]). It was reported that an increase in muscle mass upon GH treatment is followed by improved muscle strength only if the exercise is intensified (Lamberts, 2000[83]). Some up-to-date meta-analysis of randomized clinical trials suggests that GH treatment, applied to the subjects with the risk of osteoporosis, results in non-significant increases of bone mineral density (Atkinson et al., 2017[23]). Furthermore, one year of IGF-1 application to postmenopausal women, in a dose (15 μg/kg, twice a day) sufficient to elevate circulating IGF-1 to young normal values, was not effective in altering the body composition or improvement of bone density, strength, mood and memory of these women (Friedlander et al., 2001[56]). Also, GH treatment in the elderly appeared problematic for some additional reasons. It was shown that GH administration in healthy ageing subjects frequently caused dose-dependent fluid retention, insulin resistance/increased glucose, gynecomastia and carpal tunnel syndrome (Rudman et al., 1990[118]; Yuen et al., 2004[148]; Liu et al., 2007[86]; Giordano et al., 2008[62]). On the other hand, an increased cancer rate linked to the GH treatment in elderly has not been reported, probably due to short-lasting clinical trials or a specific timing of the therapy (end of the life), when there is not enough time for a cancer development (Giordano et al., 2008[62]).

Given that the hypofunction of somatotropic system in ageing mostly reflects disturbance in the neural control of GH secretion, treatment with GHRH or GH secretagogues has also been proposed in elderly people (Aimaretti et al., 2004[2]; Giordano et al., 2008[62]). Some short-lasting GHRH treatments have been shown to restore spontaneous GH secretion and IGF-1 levels in the ageing subjects (Corpas et al., 1992[42]), but there was no increase in physical performance scores upon its administration (Borst, 2004[30]). Additionally, the crucial problem of GHRH use in the clinical praxis is that it needs to be parenterally administered (Giordano et al., 2008[62]). GH secretagogues, as a synthetic peptide or non-peptide substance, may stimulate GH secretion by acting at the pituitary or at the level of hypothalamic GHRH neurons (Ghigo et al., 1999[58]). While peptidergic GH secretagogues were ineffective on the GH/IGF-1 levels in elderly people (Rahim et al., 1998[117]), some non-peptidergic analogues applied to the same population stimulated the GH secretion, that have restored the IGF-1 production at the young subject values (Aloi et al., 1994[16]). The expressed concern related to these secretagogues is whether they can significantly affect the body composition, metabolism or cognitive functions associated with ageing (Giordano et al., 2008[62]).

Keeping in mind that the somatopause coincides with low free testosterone in ageing (andropausal) men, it seems logical that the testosterone supplementation may be considered as a therapeutic strategy in the elimination of the symptoms. It was observed that transdermal testosterone application (5 mg/day, 5-6 weeks) to elderly (61-76 years old) men did not alter neither GH pulse frequency and amplitude nor the maximal GH peak (Orrego et al., 2004[110]). Judging from the fact that testosterone supplementation to healthy elderly men was not effective in the normalization of pituitary GH output, the authors concluded that age-related testosterone deficiency is unlikely to be the proximate cause of the somatopause (Orrego et al., 2004[110]). When it comes to the simultaneous treatment of ageing male with GH and testosterone, results appear to be controversial. While the certain studies suggest the lack of positive interaction between these two hormones in the impact on muscle performance (Giannoulis et al., 2006[61]), there is some evidence of significantly increased aerobic capacity/muscle mass and decreased total abdominal fat during the treatment with the combination (Blackman et al., 2002[29]; Giannoulis et al., 2012[60]; Sattler, 2013[120]). On the other hand, the estrogen replacement was suggested to affect GH responsiveness by causing relative GH resistance (Gleeson and Shalet, 2009[65]). Münzer et al. (2006[104]) observed some slightly increased IGF-1 levels in healthy aged women, upon the hormone replacement therapy (100 μg/day estradiol patch plus 2.5 mg of medroxyprogesterone acetate, for the first 10 days of each month/26 weeks). The similar pattern of estradiol treatment, alone or combined with GH, did not significantly affect a muscle strenght in healthy aged women (Blackman et al., 2002[29]).

Besides the hormone supplementation strategies in a service of the recovery of somatopausal symptoms, a certain number of pharmaceuticals are developed for that purpose. Thus, cerebral selective cholinesterase-inhibitors (rivastigmine and donepezil) were shown to be beneficial for the enhancement of GH release and the elevation of IGF-1 levels in elderly human subjects, but their long-term efficacy and safety remained questionable (Obermayr et al., 2003[108], 2005[107]). Additionally, alfacalcidol (1-hydroxycholecalciferol), which is an analogue of vitamin D, has performed the multifactorial effects on the somatopause familiar symptoms (Schacht et al., 2005[121]). The best known are its anti-bone loss effect, as well as the action on muscle power, which reduces falls and the related bone fractures (Schacht et al., 2005[121]).

In the section of this analytical text that follows, we will accentuate the effects of soy isoflavones on the somatotropic system in the experimental *in vivo* models. As above mentioned, polyphenols of natural origin, especially soy isoflavones, possess the evidence-based potential in the remediation of wide spectrum of ageing-related symptoms and diseases. Their effects at the level of morphophysiological and pathological basis of the somatopause should receive the deserved attention. Soy isoflavone abundant presence in the specific foods (tofu, miso, soy milk, soy sauce, etc.), the proportionally low cost of purified compounds, a simple formulation in the food supplements or alternative remedies and the related facilitated administration, all together additionally recommend their usage in this respect. However, their potentially endocrine-disrupting effects shouldn't be disregarded in designing the therapeutic strategy (Bennetau-Pelissero, 2016[27]).

## The Soy Isoflavone Effects within the Somatotropic System of Experimental Models

Considering the above mentioned weaknesses of existing therapeutic approaches pertinent to the somatopausal symptoms, as well as the known side effects of “classical” HRT, we believe that the research efforts should be made to identify some agents that may have a better risk/benefit profile. Currently, there is the credible opinion that polyphenols of natural origin could be a good and relatively safe replacement for the hormone therapy. A detailed mechanistic overview of soy isoflavone actions, that may be useful while reading this section, can be found in our previous review articles (Ajdžanović et al., 2014[6], 2015[7]).

In an attempt to light up the potential usefulness/weakness of soy-isoflavone supplementation when it comes to the somatotropic system in different experimental models (dominantly the ageing ones), the relevant research data is summarized in this section. Far ago was shown that genistein had enhanced GHRH-stimulated cAMP accumulation and GH release in rat anterior pituitary cells (Ogiwara et al., 1997[109]). The Trifunović et al. (2012[137], 2014[133], 2016[136]) respectable studies demonstrated the effects of soy isoflavone genistein on the analytical and quanititative histology-related parameters of somatotropic system and GH output in a rat model of the mild andropause (orchidectomized adult rats). As already stated, some modern stereological tools were used in the obtaining of results. The applied dose of genistein (30 mg/kg b.m.) was chosen to mimic human exposure to elevated concentrations of isoflavones (Doerge and Sheehan, 2002[47]). Precisely, following the genistein treatment we found an increase of the Arc nucleus volume by 24 % while Pe nucleus volume didn't change, compared to the orchidectomized control (Trifunović et al., 2016[136]). Morphological remodeling of the neurons and glial cells *via* ERs could be responsible for the larger Arc nucleus volume, considering improved neuronal cell proliferation observed in some brain regions upon genistein application *in vitro* (Pan et al., 2012[111]). Visible GHRH immunoreactivity in the Arc nucleus as well as SS immunoreactivity in the Pe nucleus were detected upon genistein treatment of orchidectomized adult rats (Figure 3c, f(Fig. 3)) (Trifunović et al., 2016[136]). Also, genistein treatment led to higher volume density of the Arc GHRH neurons by 26 % and improved GHRH intensity of staining in the ME, all compared to the control (Figure 4c(Fig. 4)), probably operating through the ER-dependent mechanisms, considering that 70 % of those neurons express ERs (Kamegai et al., 2001[79]; Trifunović et al., 2016[136]). The volume density of SS neurons was 1.5 fold higher, followed with higher SS imunofluorescence intensity in the ME (Figure 4f(Fig. 4)), all after genistein treatment, probable as the result of this isoflavone indirect action through an intraneuronal system (Herbison and Theodosis, 1993[70]; Trifunović et al., 2016[136]). At the pituitary level, genistein treatment increased pituitary weight by 28 % and the volume by 21 %, in comparison with the orhidectomy alone (Trifunović et al., 2014[133]). The volume density and total number of GH cells were 18 % and 36 % higher in genistein treated group than in the orchidectomized controls, respectively (Figure 5c(Fig. 5)) (Trifunović et al., 2014[133]). Plus, there was a tendency for the GH cell volume to be greater following treatment with this isoflavone (Figure 5c inset(Fig. 5)) (Trifunović et al., 2014[133]). The stimulating action of genistein on the stereological data within hypothalamus and pituitary has led to increased concentration of GH. Precisely, the GH blood concentration was higher by 1.3 fold in comparison with the orchidectomized control group (Trifunović et al., 2014[133]). It is evident that genistein refreshed and stimulated the somatotropic system function in a rat model of the mild andropause. In line with this, the elevation of circulating GH as well as the increase of the expression levels of the GHr in the liver tissue has been found in Sprague-Dawley rats treated (10 days) with cheonggukjang, a fermented soybean product rich in genistein and daidzein (Hwang et al., 2014[73]).

In the animal model of severe andropause (orchidectomized middle-aged rats), applied genistein or daidzein (30 mg/kg b.m.) returned the mean body mass at a level observed in sham-operated group, *i.e.* increased its value in comparison with the control group where only the orchidectomy was performed (Ajdžanović et al., 2014[8]). After daidzein treatment, the absolute and relative pituitary weights were increased by 8.4 % and 15 % respectively, while in genistein treated group the relative pituitary weight increased by 24 %, all compared to the orchidectomized control group (Ajdžanović et al., 2014[8]). The main characteristic of GH cells in genistein or daidzein treated rats is a weaker intensity of immunospecific staining compared to the orchidectomized group, while the pituitary capillaries, towards which GH cells gravitate, are dilated (Figure 6c, d)(Fig. 6). Relative intensity of fluorescence within the pituitary GH cells was decreased by 44.8 % and 50 %, following genistein and daidzein treatment, respectively (Figure 7c, d(Fig. 7); Reference in Figure 7: Ajdžanović et al., 2014[8]). Also, the volume of GH cells was smaller by 13.8 % and 11.9 %, while their relative volume density was decreased by 65.4 % and 64 %, all following genistein and daidzein treatments respectively (Figure 6c, d(Fig. 6)) (Ajdžanović et al., 2014[8]). Also, our findings indicate that daidzein treatment stimulated thyroid C cells, increased trabecular bone mass and decreased bone turnover in the same animal model (Filipović et al., 2010[50]). Low dosed genistein or daidzein (10 mg/kg b.m.) were shown to increase TSH, decrease thyroid hormone circulating levels as well as to increase the peripheral tissue availability of thyroid hormones, all when applied in the animal model of severe andropause (Šošić-Jurjević et al., 2010[127], 2014[129]). The cholesterol (total, LDL and HDL) blood concentrations significantly decreased, but the total triglyceride circulating concentrations increased, upon low (10 mg/kg b.m.) and high (30 mg/kg b.m.) genistein or daidzein application to orchidectomized middle-aged rats (Šošić-Jurjević et al., 2007[126]). Appearance of the somatotropic system and the related biochemical/physiological parameters, reflecting the soy isoflavone application in the animal model of severe andropause, are not all in the perfect coherence when it comes to the potential benefit of the substances' supplementation. Namely, the observed triglyceride concentrations increase call for some precautions, while the GH blood concentration should be measured for the purpose of confirmation of intensified GH cell-hormonal content secretion.

It should be emphasized that intracerebroventricular (ICV) infusion of genistein (40 μg/400 μl/4 h), applied to ovariectomized ewes six weeks after the surgery, increased the mean plasma GH concentrations in comparison with the control values (Misztal et al., 2007[101]). Results from the same study showed the decreased values of GH-positive cells percentage as well as the GH immunostaining density, suggesting diminished hormone storage and its intensified secretion (Misztal et al., 2007[101]). Obviously, in an animal model represented by estradiol-deprived females (like in menopause), genistein stimulated the GH output, which from the musculoskeletal point of view may be considered as a positive outcome. Also, the stimulation of the amplitude of GH pulses was observed by the same research group in the rams ICV treated with genistein (total 40 μg) (Misztal et al., 2008[100]).

Finally, some studies show the effects of genistein on the somatotropic system that should be also taken into account. Thus, the study on the Nile tilapia fish (*Oreochromis niloticus*) was conducted to examine the effects of dietary genistein on the endocrine disruption on GH/IGF-1 system (Chen et al., 2016[39]). There weren't changes in the plasma GH and IGF-1 levels in fish fed with diets containing 30 μg/g and 300 μg/g genistein, while the mRNA expression of genes along the GH/IGF-1 system remained unaffected (Chen et al., 2016[39]). In the fish fed with 3000 μg/g genistein diet, the plasma GH and IGF-1 levels decreased, and the mRNA expression of GH and GHr2 was depressed (Chen et al., 2016[39]). This study provides convincing evidence for the growth impediment after high-dosed genistein application, by disturbing the GH/IGF-1 axis, in Nile tilapia *Oreochromis niloticus* (Chen et al., 2016[39]).

To the best of our knowledge, the changes of somatotropic system upon soy isoflavone application haven't been clinically monitored, yet. In addition to beneficial bone- and metabolism-related clinical findings, pertinent to their usage (Zhuo et al., 2004[149]; Messina, 2010[93]; Jackson et al., 2011[75]; Cavallini et al., 2016[36]), this specific aspect deserves a greater attention.

## Conclusion

From the previous detailed elaboration of somatopause phenomenology and the weaknesses/uncertainties related to existing therapeutic approaches, it is clear that there is a need for discovering some more effective, and at the same time safe alternatives. It seems that soy isoflavones, potentially cheap substances whose easy application shows a number of benefits when it comes to the wide spectrum of ageing symptoms, may play a certain role in this context. At least, the presented data from experimental ageing studies, which necessarily need to be preclinically and clinically filtered regarding the somatopausal symptoms, hint some cautious optimism and call for further designing of balanced soy isoflavone-based therapeutics.

## Acknowledgements

This work was supported by the Ministry of Science, Education and Technological Development of the Republic of Serbia, Grant number 173009. Figures 3-7(Fig. 3)(Fig. 4)(Fig. 5)(Fig. 6)(Fig. 7) are adopted from our previous publications and reprinted by permission of the Licensors - publishers Taylor & Francis (Trifunović et al., Nutritional Neuroscience 19: 467-474, 2016[136]), Springer (Trifunović et al., Endocrine 47: 869-877, 2014[133]) and Faculty of Veterinary Medicine, University of Belgrade, Serbia (Ajdžanović et al., Acta Veterinaria - Beograd 64: 93-104, 2014[8]). Appropriate citation is provided in the Figure legends while full references are listed at the Reference list, according to EXCLI Journal Instructions to Authors. The authors Vladimir Ajdžanović and Verica Milošević are participating to the COST Action FA 1403 POSITIVe (Interindividual variation in response to consumption of plant food bioactives and determinants involved), supported by COST (European Cooperation in Science and Technology). We are grateful to Mr. Hinko Sauter (SWISS CONCEPT SCIENCES D.O.O., Belgrade, Serbia) for additional technical support.

## Conflict of interest

The authors declare that they have no conflict of interest.
